# Dispersion Characteristics of Hazardous Gas and Exposure Risk Assessment in a Multiroom Building Environment

**DOI:** 10.3390/ijerph17010199

**Published:** 2019-12-27

**Authors:** Xiaoping Liu, Zhen Peng, Xianghua Liu, Rui Zhou

**Affiliations:** 1 College of Civil Engineering, Hefei University of Technology, Hefei 230009, China; liuxp@hfut.edu.cn (X.L.); zpeng328@163.com (Z.P.); liuxianghua@hfut.edu.cn (X.L.); 2 Institute of Public Safety Research, Department of Engineering Physics, Tsinghua University, Beijing 100084, China

**Keywords:** accidental leakage, multiroom building, concentration distribution, risk assessment, computational fluid dynamics (CFD)

## Abstract

The leakage of hazardous chemicals during storage and transport processes is a kind of commonly occurring accident that can pose a serious threat to people’s lives and property. This paper aims to investigate the airflow and dispersion characteristics of hazardous gas around a multiroom building, and evaluate the corresponding exposure risks. The effects on indoor air quality (IAQ) when polluted air enters a room under different indoor and external conditions were examined by using a computational fluid dynamics technique. First, the numerical model established herein was verified by the available wind-tunnel experimental data, and acceptable agreement was found between the predicted and measured velocities. Subsequently, the effects of different natural ventilation paths, wall porosities and outdoor pollutant source characteristics on the airflow and contaminant distribution were evaluated. The study not only reveals the airflow pattern and concentration distribution in indoor spaces under different natural ventilation conditions but also quantitatively analyzes the relationship between the probability of death and the corresponding source strength under the circumstance of pollutant leakage near a building. The results can be useful for the prevention and control of hazardous chemical gas leakages and provide some guidance on evacuation after an accidental or routine leakage.

## 1. Introduction

In recent years, the dispersion of pollutants in different building environments due to either accidental or routine releases has become a typical problem in environmental protection issues [1,2,3]. With the development of industry, large amounts of dangerous substances are transported by road and rail, and transport accidents of dangerous substances are increasingly frequent. Gaseous hazardous chemical leakage during storage and transport processes is a common accident [4,5]. Once the leakage of hazardous chemicals happens during transport process, the released toxic gases generate cloud in street canyons and can cause huge disaster through their physical or chemical properties. Although the majority of incidents involving the release of hazardous gas affect outdoor environments, the gas from such incidents can also diffuse indoors through the intake of ventilation systems or any kind of openings when the pollutant source is near a building and possibly cause serious consequences for the occupants [6]. Many industrial accidents result from the accidental release of hazardous gases. Bernatik et al. [7] reviewed the results of different methods of modelling releases and dispersion of dangerous gases in cases of major accidents from road and rail transportation. One of the most severe cases was the release of H_2_S in the town of Gao-qiao, Kai Xian, China on December 23, 2003, which caused 243 deaths and the evacuation of approximately 64,000 residents [8]. In the Viareggio liquefied petroleum gas (LPG) accident, the overturned tankcar led to the release of about 46.7 t of LNG, which resulted in 32 fatalities [9].

Traditionally, natural ventilation is generally considered to be an energy efficient alternative for providing a satisfactory indoor air environment, and occupants are accustomed to opening windows during transition seasons. However, if the leakage source is near a building, indoor personnel can be exposed to the risk of death, and the magnitude of the risk will be different under different indoor and external conditions. Thus, it is of great significance to study the transmission characteristics of pollutants under different ventilation paths for the assessment and prevention of hazards and the evacuation of personnel.

Many scholars have researched airflow and pollutant dispersion in buildings with wind-driven natural ventilation and shown that the main factors affecting the air flow and pollutant concentration distributions are the approaching wind (speed and direction), building openings (size, shape and position), pollutant sources and ventilation paths [10,11,12,13,14,15,16,17,18]. For example, Nikas et al. [10] numerically predicted the flow patterns around and inside a naturally ventilated building and revealed the importance of both the incidence angle and the speed of the approaching wind on the ventilation rate of that building. Shetabivash [11] investigated the effects of the opening position and shape on the airflow pattern inside a building. Chu et al. [12] conducted wind tunnel experiments to investigate wind-driven ventilation in buildings with two openings on a single wall and measured the air exchange rate by the tracer gas decay method under different wind speeds, directions and opening sizes. Mavroidis et al. [13] studied the plume dispersion around a single obstacle by field and wind tunnel measurements and considered the effects of the discharge position of the pollutant source, the angle of the approaching wind and the building configuration. Kao et al. [14] numerically investigated the airflow and particulate matter (PM) transport characteristics in multiroom buildings under four typical natural ventilation patterns with the same air change rate. Liu et al. [15] investigated different ventilation modes and source location effects on indoor air pollution dispersion in a zoned room using CFD method. Di et al. [16] studied the effect of source location under single-sided natural ventilation and cross natural ventilation on inter-flat pollutant transmission characteristics in a multi-room building using a 1:30 scaled model. Chung and Hsu [17] investigated the ventilation efficiency of different ventilation patterns arranged by two inlet and two outlet diffusers at different locations. Lo and Novoselac [18] demonstrated the dynamic nature of wind driven cross ventilation flow in a multi-zone building by illustrating four kinds of ventilation path. These previous studies illustrated that the flow and air contamination distribution can be obviously different under different ventilation paths, especially for a multiroom building configuration. It is therefore important to investigate the effect of ventilation path and quantitatively analyze the differences of indoor airflow and contaminant distribution under various indoor ventilation paths.

The above studies also provide a great deal of useful information and experience for the study of air flow distribution and pollutant dispersion inside and outside of buildings, but only a few studies have been carried out to study the effects of outdoor pollutants on the indoor environment [19,20]. Chang et al. [19] focused on the effects of the air change rate, the indoor airflow pattern, and the outdoor traffic pollution dispersion on the IAQ of a naturally ventilated building by analyzing various roof vent openings, side-vent openings, and outdoor wind speeds. Tong et al. [20] employed a CFD-based air quality model to quantify the impact of traffic-related air pollution on the indoor air quality of a naturally ventilated building and found that the indoor particle concentrations strongly depend on the distance between the roadway and the building, the particle size, the wind conditions and the window location.

Risk assessment after the leakage of a pollution source is also a hot topic. The combination of a CFD tool and a risk assessment model, mainly including the dose-response model [21,22,23] and the Wells-Riley model [24,25], has been widely used in recent years. For example, Pontiggia et al. [21] assessed the consequences of hazardous gas release in urban areas through CFD modeling. Zhang et al. [22] combined a CFD numerical simulation and the dose-response model to propose a quantitative analysis of acute toxic gas exposure threats. First, they set up and solved the CFD equations to acquire the real-time concentration field of toxic gas release and dispersion and then calculated the toxic dose according to the gas concentration and exposure time. Lastly, they estimated the number of expected fatalities using the dose-response model. With respect to assessing the infection risk, Qian et al. [24] integrated the Wells-Riley equation into a CFD model and evaluated the infection risk of the airborne transmission of diseases in a hospital ward.

These aforementioned studies focused on the death or infection probability in different areas after the source leak of pollutants but did not study the impact of the source strength on the death probability of different indoor areas. Moreover, the indoor airflow and pollutant dispersion in a multiroom building can be quite different from those in a single-room building. 

In summary, this paper chooses a scenario in which H_2_S is accidentally leaked around a single-story multiroom building and numerically studies the air flow and pollutant concentration distribution in each room under different natural ventilation paths and outdoor pollutant source locations. The presented case scenario is based on a hypothetical multiroom building under natural ventilation condition, thus the IAQ level is strongly depending on outdoor air condition. Once there is an accidental release of H_2_S around the building, the gaseous pollutants are likely to disperse around the building and enter the indoor environment through the opening under natural ventilation condition. The IAQ of the target building will be significantly affected and the occupants may be exposed to huge risk. The differences under various scenarios are quantitatively analyzed. By employing the dose-response model, the relationship between the source strength and the probability of death in different areas of a building under different ventilation paths and pollutant positions is studied. The effects of different wall porosities on indoor airflow and pollutant distribution are also considered.

## 2. Model Validation

### 2.1. Description of the Wind-tunnel Experiment

Karava et al. [26] used the wind tunnel and particle image velocimetry (PIV) technique to investigate the air velocity field in a cross-ventilated building. The experimental study was carried out in a boundary layer wind tunnel at Concordia University (Montreal, QC, Canada). The wind tunnel was 12 m in length, 1.8 m in height and 1.8 m in width.

**Figure 1:**
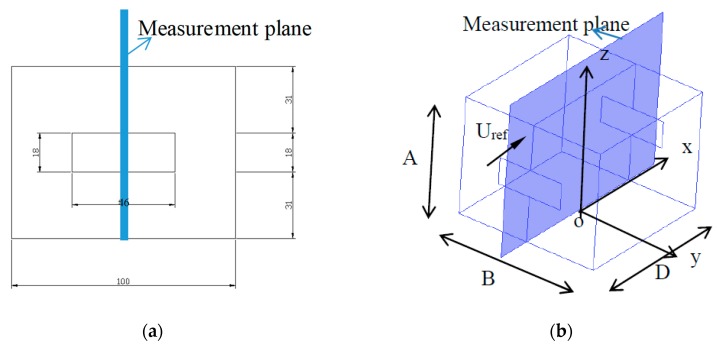
(**a**) Front view of scaled model with opening size and dimensions; (**b**) Measurement plane for the configuration of 10% wall porosity.

Building models with different openings and a 1:200 scale were built from 2 mm cast transparent polymethylmethacrylate (PMMA) sheets. The length, width and height of the scale building were 100 mm, 100 mm and 80 mm, corresponding to actual dimensions of 20 m, 20 m, and 18 m, respectively. The mean velocity and turbulence intensity were measured by a hot-wire probe in selected locations, and the PIV data were obtained in the vertical symmetry plane across the centerline of the opening position. In the experiment, the openings were at the center of the two opposite walls, and different wall porosities (A_opening_/A_wall_) were obtained by changing the window width. More specific experimental details can be found in the studies by Karava et al. [26]. The experimental results with a 10% wall porosity were selected to verify the reliability of the numerical simulation. The structure of the building and the measurement plane for comparison are shown in Figure 1.

### 2.2. CFD Simulation Settings

Owing to the great advancement in computer science, the accuracy and reliability of CFD computation have improved substantially, and it has been widely used to study real-life problems, including both gas dispersion study and exposure risk assessment [27,28]. It is commonly recognized that CFD simulations can be very sensitive to a large number of computational parameters. Validation of the accuracy and reliability is the prerequisite before employing CFD method to study real-life problems [29]. Comparing wind tunnel experimental data with simulation results is a normally used method to verify the reliability of the CFD model [30]. To demonstrate the reliability of the numerical methods, a 1:1 numerical model was established, and the simulation results were compared with the experimental data. The commercial CFD code FLUENT 15.0 which is based on the finite volume method (FVM) is used to solve the steady-state isothermal flow field. The SST k−ω turbulence model presented by Menter [31] is used in the present study. Compared with the k−ε model, the k−ω model is considered to be more accurate in predicting free shear flows and has been recommended for predicting wind-induced airflow in and around buildings [32].

**Figure 2:**
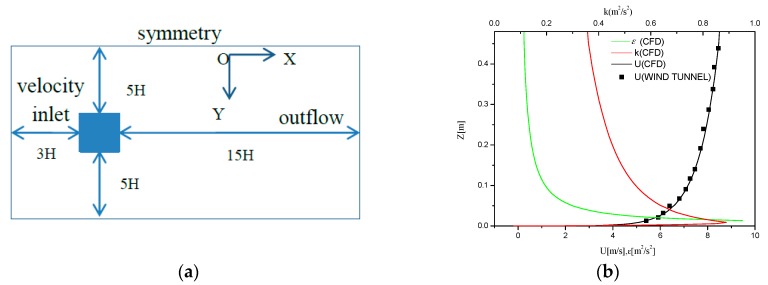
(**a**) Computational domain (top view); (**b**) Boundary conditions used in CFD simulations and wind tunnel experiments.

Based on the best practice guidelines summarized by Franke [33] and Tominage et al. [34], an appropriate computational domain was established, as shown in Figure 2a. H represents the building height, while the entrance was set as a velocity inlet located 3H in front of the building to limit the development of unintended streamwise gradients. The outflow boundary condition was imposed at the outlet plane, which was 15H behind the building. Slip wall conditions, i.e., zero normal velocity and zero normal gradients of all variables, were set at the top and lateral sides of the domain, which were 5H away from the building roof and sidewall, respectively. The approaching mean wind speed profile in the wind tunnel test can be described by the logarithmic law in Equation (1), where $u_{\mathrm{ABL}}^{*}$ is the friction velocity, κ is the von Karman constant, which is equal to 0.42, and $z_{0}$ is the aerodynamic roughness length, which is equal to 0.025 mm (reduced scale). The turbulent kinetic energy (k) was calculated from the mean wind speed and the measured intensity ($I_{u}$) using Equation (2), while the coefficient was set to 1 for the validation case. The turbulence dissipation rate (ε) was given by Equation (3), and the specific dissipation rate (ω) was given by Equation (4), where $C_{\mu }$ is an empirical constant equal to 0.09. Both the velocity profile and the kinetic energy of turbulence were imposed at the domain inlet in the CFD simulation as it was used in the wind tunnel, as shown in Figure 2b.

(1) $$U\left(z\right)=\frac{u_{\mathrm{ABL}}^{*}}{\kappa }\ln\left(\frac{z+z_{0}}{z_{0}}\right)$$

(2) $$k\left(z\right)=a{\left(I_{u}\left(z\right)U\left(z\right)\right)}^{2}$$

(3) $$\varepsilon \left(z\right)=\frac{u_{\mathrm{ABL}}^{*3}}{\kappa \left(z+z_{0}\right)}$$

(4) $$\omega \left(z\right)=\frac{\varepsilon \left(z\right)}{C_{\mu }k\left(z\right)}$$

The building walls were set to no-slip walls. Based on the findings of Blocken et al. [35] (Equation (5)), the values of the sand-grain roughness height $k_{s}$ (m) and the roughness constant $C_{s}$ were determined through the consistency relationship with the aerodynamic roughness length. In addition to the ground surface (where $k_{s}\;$= 0.28, $C_{s}\;$= 0.874), other building surfaces were modeled with a zero roughness height ($k_{s}\;$= 0). The SIMPLE algorithm was used for pressure-velocity coupling, and second-order discretization was used for both the convection terms and the viscous terms of the governing equations. Convergence was considered to be obtained when the scaled residuals tended to be stable and reached a minimum value of 10^−4^ for all the variability:

(5) $$k_{s}=9.793z_{0}/C_{s}$$

### 2.3. CFD Validation: Grid Independent Tests and Comparisons

**Figure 3:**
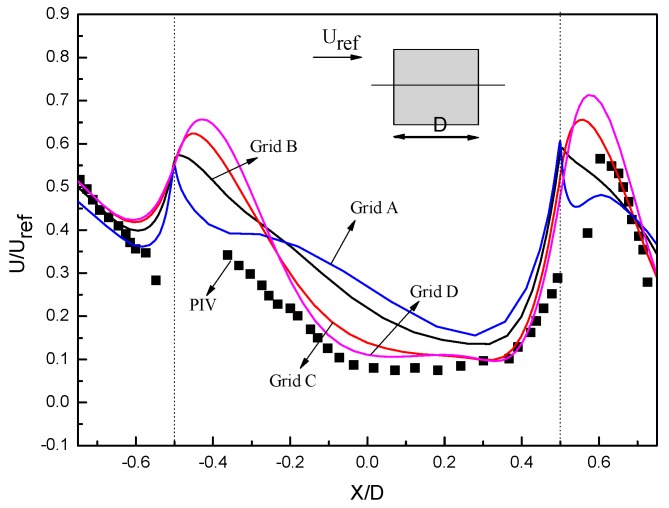
Grid sensitivity analysis and comparison between experimental and simulation results for the validation case.

The non-uniform hexahedral mesh was used here and the stretching ratio was kept under 1.2 in the surroundings of the building model. Four grid arrangements, Grid A (530,000 cells), Grid B (1,040,000 cells), Grid C (2,050,000 cells) and Grid D (3,100,000 cells), were made by changing the number of nodes and the distance from the center point of the wall adjacent cell to the wall, as shown in Figure 3. It can be clearly seen that the numerical simulation results under Grid C and D are similar and show better agreement with the experimental results. Considering the calculation cost and numerical precision, the same grid arrangement method as that used with Grid C was selected for comparison and additional studies. Cells were adapted near the wall to be no larger than 0.01 mm, and the value of y^+^ is under 5. These values ensure that the center point of the wall-adjacent cell is located in the viscous sublayer, which satisfies the requirement of the k−ω model.

Figure 3 also depicts a comparison between the experimental (PIV) measurements and the numerical (CFD) results in terms of the normalized streamwise wind speed along the centerline. The model is able to accurately capture the acceleration both near to the openings and inside the building, while was less accurate in the immediate vicinity of the opening. It is because the effects of shadows or reflections could have caused uncertainties in the PIV measurements at these positions [26,36]. With the limitation in turbulence modeling, the numerical results in the recirculation regions are more prone to errors, which also contributes to large discrepancies. Overall, the performance of the SST k−ω model could be considered acceptable. The acceptable agreement between the PIV and CFD datas validates the capability of the SST k−ω model to simulate the wind-driven cross ventilation.

## 3. Configuration Descriptions

### 3.1. Model Setup

**Figure 4:**
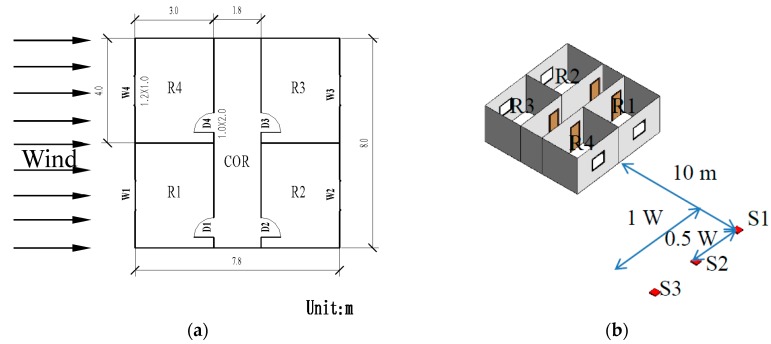
(**a**) Schematic diagram; (**b**) Plan view of the building model.

Based on the validated numerical method, a numerical model is established to study the effects of the ventilation path and outdoor source location on the indoor airflow and concentration distribution in a multiroom building. The model represents a slab-shaped multiroom building, which is a very common and basic structure used in hotels, apartments, student dormitories, hospital wards, etc. The model has a common corridor separating the two sides, each of which has a flat façade with openable windows. Figure 4a presents the model size details and the location of each opening. The dimensions of the model are L × W × H = 7.6 m × 8.0 m × 3.0 m, with four rooms symmetrically distributed on both sides of the corridor. The size of each single room is L × W × H = 3.0 m × 4.0 m × 3.0 m, and each room contains one window and one door. The window is opposite to the door. The width and height of all the doors are 1.0 m and 2.0 m, respectively. Four windows are located in the middle of the corresponding external walls and have the same size of 0.6 m × 1 m or 1.2 m × 1 m, corresponding to a wall porosity of 5% or 10%, respectively.

Considering both the prediction accuracy and the computational costs, a 1:100 scale model was chosen for the following simulation. For the investigation of airflow and pollutant dispersion in the reduced-scale model, one set of requirements for similarity between the scale model and the prototype should be carefully examined, as reported in several studies [37,38,39]. Within these similarity conditions, several of the dimensionless parameters can be neglected due to their low relative importance, while the Reynolds number (Re) independence is required, which is one of the most important criteria for the study of isothermal flow and pollutant dispersion fields in buildings. According to the Re-independence theory, when the Re number exceeds a critical value, the flow field would enter a Re-independent regime, and the flow characteristics do not change with the increase of Re [40]. The calculation of the Re number for a building is based on the building height (H) and the reference wind velocity at the building height ($U_{\mathrm{ref}}$), defined as $R_{e}=U_{ref}H/v$, where *v* is the kinematic viscosity. In this paper, the reference velocity at the building height ($U_{\mathrm{ref}}$) is 5.53 m/s and obeys a building Reynolds number of 1.1 × 10^4^, which is higher than the previously presented critical value to obtain Reynolds-number independence [38,41].

H_2_S is a hazardous chemical which often leads to accidents due to its leakage [8,22]. This paper chooses a scenario in which H_2_S is accidentally leaked during transportation around the building, and the leakage source is located 10 meters away from the windward side of the building, with different lateral displacement locations (y = 0 W, y = 0.5 W, y = 1 W), as shown in Figure 4b. Gaseous pollutant is used and is released from different source locations at a constant flow rate. The H_2_S density is 1.46 kg/m^3^ and the air density is 1.225 kg/m^3^. In this study, the dispersion characteristics of H_2_S were studied while the chemical reaction during the dispersion process was ignored. The density difference between H_2_S and air is taken into consideration in this study. The density of mixture is calculated by volume-weighted-mixing-law in the presented CFD simulations. The pollutant release velocity is low enough to ensure that source momentum effects are not significant. Britter’s criterion [42] (Equation (6)) is applied to ensure that the release is passive, where d (30 mm, in the prototype) is the equivalent diameter of the release source, q is the tracer gas volumetric flow rate, and g’ is the gravity modified by the density difference between the tracer and the air, given by $\;g\;\prime =g\left(\left|\rho -\rho_{a}\right|/\rho_{a}\right)$. This paper chooses a typical value of 2.25 × 10^−3^ m^3^/s (in the prototype) as the volumetric gas flowrate, and the value of the parameter is 0.08 for the H_2_S source, according to Equation (6).

(6) $$\frac{{\left(\;g\;\prime \;q/d\right)}^{1/3}}{U_{\mathrm{ref}}}\leq 0.15$$

**Figure 5:**
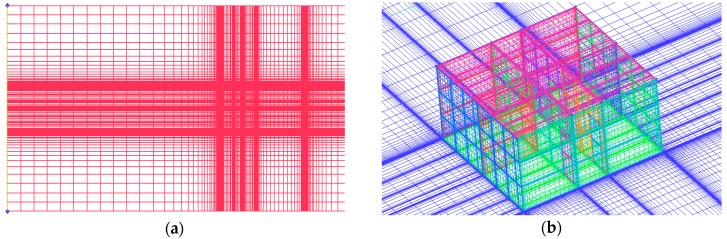
(**a**) The overall grid schematic diagram (top view); (**b**) Local grid schematic diagram of the building surface.

The details of the numerical simulation method are the same as those used in previous validation cases. The total number of computational cells is 1981980, and Figure 5 shows the detailed arrangement of the mesh. When the concentration equation is solved, the turbulent Schmidt number (Sc_t_) of 0.7 is used for the k−ω model. The airflow in the computational domain is assumed to be isothermal, and the adiabatic wall condition is applied in all the building surfaces. It took about 20 hrs to run a full case which was performed on a HP-Z820 workstation. 

**Figure 6:**
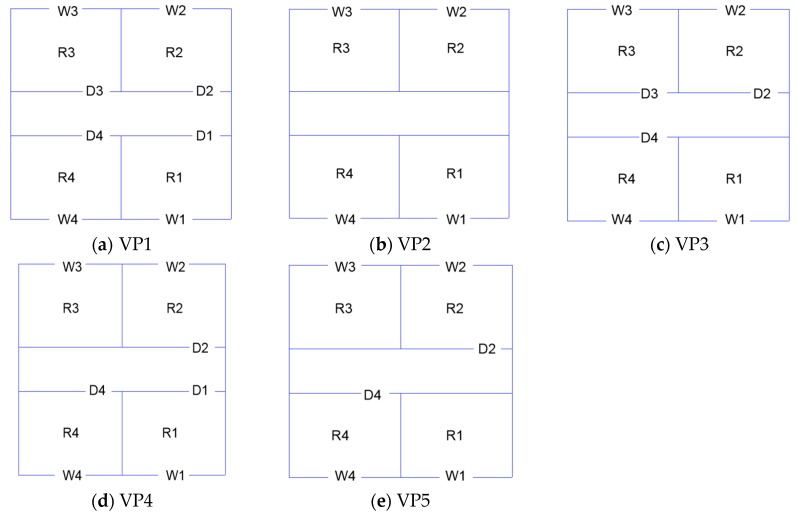
Five typical ventilation paths. (**a**) Ventilation path 1 (VP1); (**b**) Ventilation path 2 (VP2); (**c**) Ventilation path 3 (VP3); (**d**) Ventilation path 4 (VP4); (**e**) Ventilation path 5 (VP5).

The effects of the natural ventilation path (five modes as shown in Figure 6), wall porosity and pollution source location on the airflow and contaminant distribution are investigated. The detailed setups for the different cases are listed in Table 1.

### 3.2. Data Analysis Methods

The ventilation rate (Q, m^3^/s) is calculated by the integral of the velocity at the inlet opening. The calculation methods are expressed as Equations (7) and (8) for the single-sided ventilation room and the cross-ventilated room, respectively:

(7) $$Q=0.5\int \left|U_{i}\right|{\mathrm{dA}}_{W}$$

(8) $$Q=\int \left|U_{i}\right|{\mathrm{dA}}_{W}$$

where $U_{i}$ denotes the streamwise velocity magnitude, and $A_{W}$ is the area of the window.

The ventilation rate is presented in normalized form $Q^{*}$, and the pollutant concentration is presented in normalized form $K_{c}$. The expressions are as follows:

(9) $$Q^{*}=Q/U_{\mathrm{ref}}A_{w}$$

(10) $$K_{c}=\left({\mathrm{CU}}_{\mathrm{ref}}H_{\mathrm{ref}}^{2}\right)/q$$

where C, ppm, is the average concentration of the plane at z = 1.2 m (representing a person’s breath height when sitting down) in each room of the multiroom building.

To quantify the influence of the source strength on the lethal probability in humans, the dose-response model is quoted here and can be defined as Equations (11) and (12):

(11) $$V=\int_{t_{o}}^{t_{end}}C^{n}dt$$

(12) Y=a+blnV

where V represents a toxic dose; n, A, and B are constants, and a = −31.42, b = 3.008, n = 1.43 for H_2_S [43]; t is the exposure time, min; and the probability variable Y is related to the lethal probability P by Equation (13) [44]:

(13) $$P=\frac{1}{\sqrt{2\pi }}\int_{-\alpha }^{Y-5}e^{-(\frac{x^{2}}{2})}dx$$

## 4. Results

### 4.1. Non-dimensional Ventilation Rate

**Figure 7:**
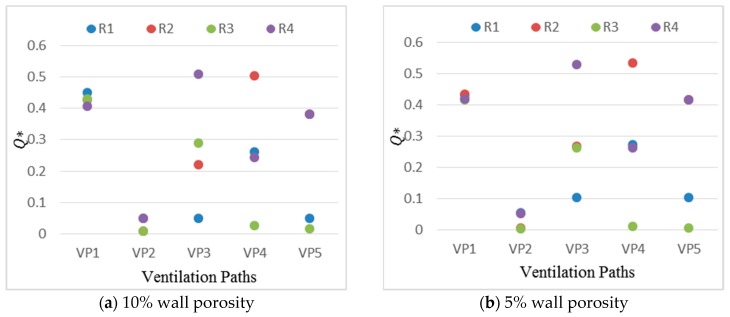
Non-dimensional ventilation rate ($Q^{*}$) in each room under five ventilation paths with two types of windward wall porosities. (**a**) 10% wall porosity; (**b**) 5% wall porosity.

The ventilation rate is an important factor that affects the distribution of indoor airflow. The ventilation rate discussed in this paper is normalized by the reference velocity at the building height multiplied by the corresponding windward opening area. Figure 7 presents the normalized ventilation rate in each room under five ventilation paths with different wall porosities. Under the same ventilation path, a similar trend can be observed with 5% and 10% windward wall porosities. Comparing different rooms, the relative variation ratio of the normalized ventilation rate in R1 is the largest, especially under VP3 and VP5, and the variation can be up to 1 time because R1 is in single-sided natural ventilation mode under VP3 and VP5, and the airflow rate entering this room is more affected by the window size compared to the cross-ventilated rooms. In addition, compared with R3 under VP2, VP4 and VP5, or R2 under VP2, which have only one window on the leeward wall for the air exchange caused by the backflow, R1 is located on the windward side. Overall, the variation of the dimensionless ventilation rate under 5% and 10% wall porosities is within an acceptable range and is not significant. Therefore, only the indoor airflow field under a 10% wall porosity is discussed in the following analysis.

The influence of different ventilation paths on the $Q^{*}$ value of each room can be clearly seen in Figure 7a. Generally, the room under cross-ventilation conditions has a much larger $Q^{*}$ value than that of under single-sided ventilation conditions, while the room located in the windward side also has a larger $Q^{*}$ value than that of in the leeward side room regardless of the ventilation path. The differences in each room under various ventilation paths show that the ventilation mode greatly affects the ventilation rate in each room.

### 4.2. Velocity Distribution under Five Ventilation Paths

**Figure 8:**
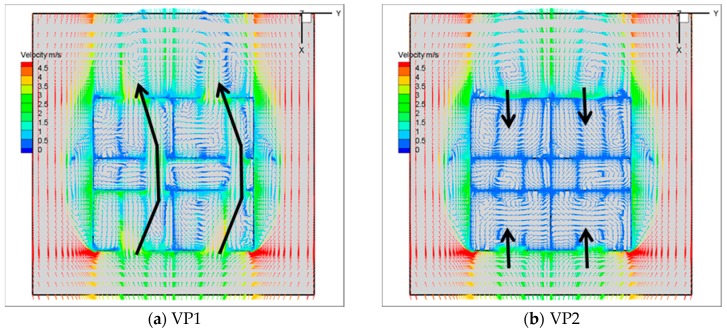

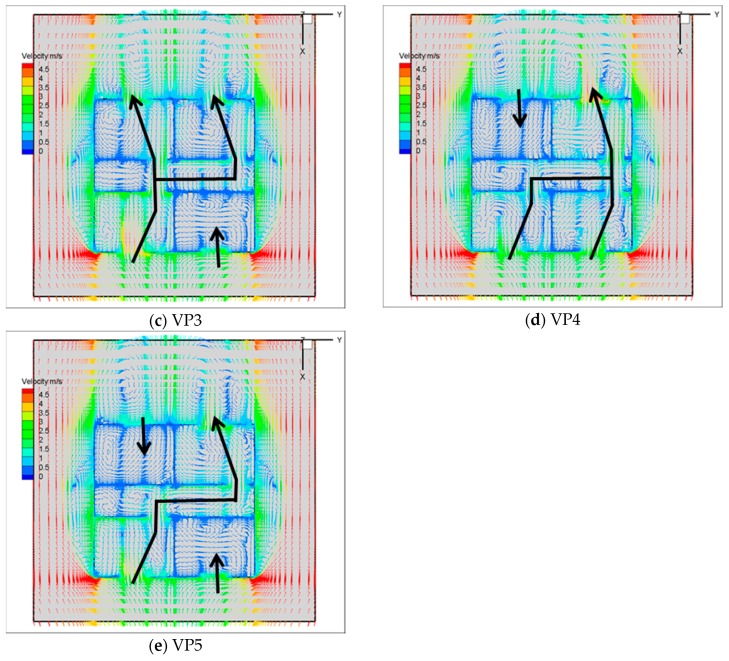
The velocity distribution at the z = 1.2 m cross-section with a 10% windward wall porosity. (**a**) VP1; (**b**) VP2; (**c**) VP3; (**d**) VP4; (**e**) VP5.

Figure 8 shows the velocity distribution and the airflow patterns in the building under five ventilation paths. It can be clearly seen that the airflow fields are quite different under various ventilation modes. The approaching wind enters the building through different openings, forming several vortices in the corridor and in each room. Under VP1, which represents the best scenario, the inflow streams coming from the windward windows are restricted to flow towards the leeward ones so that the airflow behaves like a piston flow in that region. All of the rooms in the building can receive sufficient airflow exchange and develop complete cross ventilation airflow paths from the inlet to the outlet. Under VP2, which represents the worst scenario, the airflow pattern in each room is referred to as single-sided ventilation with all the windows opened and all the doors closed. The airflow in the leeward room is extremely weak because the air exchange is only induced by the backflow through the leeward windows. There is a large low-speed area under VP2, and the well-mixed zone is the smallest under this situation.

Except for the best and worst scenarios discussed above, under VP3, as shown in Figure 8c, which has two outlets and only one inlet, the location of the inlet is the main factor affecting the airflow pattern inside the building. The air enters R4 and then flows towards R2 and R3, with total inflow rates of 37.8% and 62.2%, respectively. The position of the inlet will affect the airflow distribution in R2, and the low-speed vortices in the middle of the corridor will disappear compared to VP1. Under the ventilation paths of the single-outlet opening, as shown in Figure 8d,e, the outlet wind speed under VP4 is higher than that in VP5, which can be explained by the principle of mass conservation: when the area of the outlet is the same, the inlet air volume is larger, and the outlet wind speed is higher. Based on the comparisons between VP4 and VP5, the single-outlet ventilation path also affects the airflow distribution in the wake area of the building. The airflow distribution on the leeward side changes from two symmetrical vortices to three vortices, and the third vortex is located in the middle of the leeward wall. The greater the outlet wind speed is, the larger the area of the vortex will be. This could affect the incoming airflow of the wake area into R3, and then affect the pollutant distribution in R3.

### 4.3. Impact of the Ventilation Path on the Concentration Field

**Figure 9:**
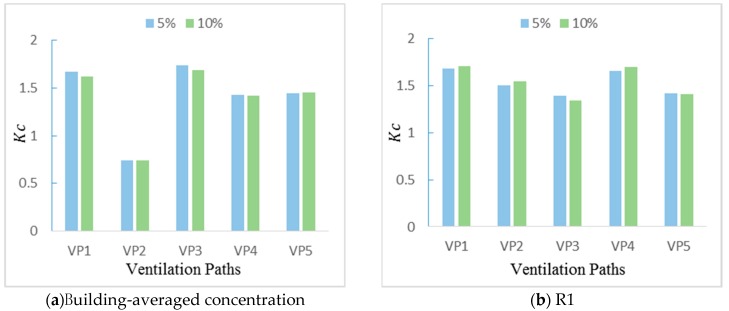

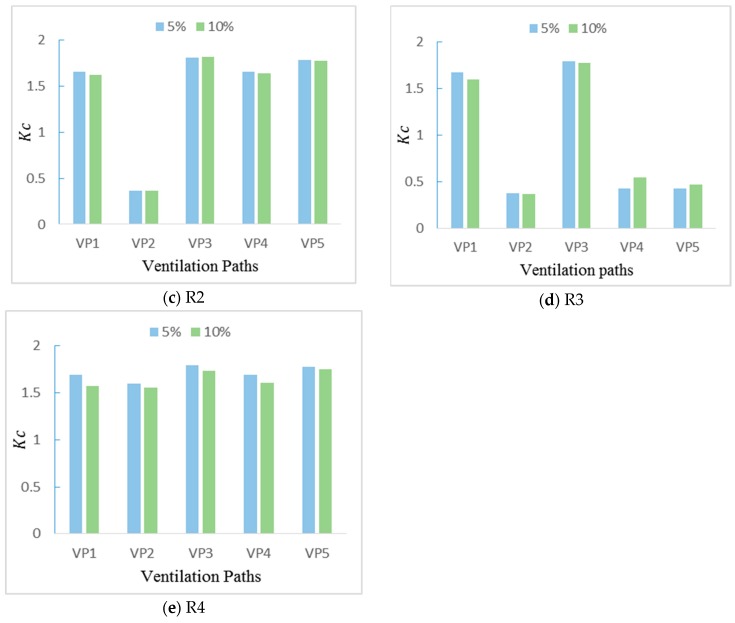
Non-dimensional concentration ($K_{c}$) at the z = 1.2 m cross-section in each room under five ventilation paths. (**a**) Building-averaged concentration; (**b**) R1; (**c**) R2; (**d**) R3; (**e**) R4.

The normalized mean concentration values at the z = 1.2 m cross-section in each room under five ventilation paths are presented in Figure 9. It can be observed that the wall porosity has a small effect on the indoor pollutant concentration when the pollutant source is leaked at y = 0 W. The concentration field at this height is basically not affected by the window area in the presented cases because the indoor pollutant concentration does not necessarily increase as the air change rate increases, which is also revealed in the study by Chang [19]. As shown in Figure 9a, the nondimensional building-averaged pollutant concentration values under double-outlet ventilation paths (VP1, $K_{c}\;$= 1.62; VP3, $K_{c}\;$= 1.69) are higher than single-outlet ventilation paths (VP4, $K_{c}\;$= 1.42; VP5, $K_{c}\;$= 1.45). The highest building-averaged concentration value under VP3 ($K_{c}\;$= 1.69) in the multiroom building is approximately 1.2 times higher than the lowest building-averaged concentration value under VP2 ($K_{c}\;$= 0.77). This result shows that a better ventilation condition is likely to introduce more outdoor pollutants into a room and lead to cross-contamination in the multiroom building when the doors and windows are always kept open. Under VP2, the pollutant concentration in the windward side rooms is approximately three times higher than that in the leeward side room, as shown in Figure 9b,c or Figure 9d,e, because the windward inlet is more conducive to the entry of the pollutant, while the appearance of pollutants in the leeward side room is due to the entrainment of the building wake. In addition, as shown in Figure 9c,e, the differences in the concentration value in each cross-ventilated room (R2 and R4) could be negligible, regardless of the ventilation path, because each room is well ventilated under this condition, which enables the pollutant to be easily transferred to different rooms.

As for different rooms, the pollutant concentration values in both R2 and R4 under single-inlet ventilation modes (VP3, VP5) are approximately 10% higher than those under double-inlet ventilation modes (VP1, VP4), as displayed in Figure 9c,e. The rooms under the single-inlet condition are less ventilated, and the increasing number of vortices lead to the pollutant being easily accumulated. For R3, the pollutant concentration values under the single-outlet ventilation paths (VP2, VP4 and VP5) are also quite different, as shown in Figure 9d. The room-averaged concentration in R3 leads to the following ranking from the lowest to the highest: under VP2 (taken as the reference value), under VP5 (27.0% higher) and under VP4 (45.9% higher). Combined with the previous velocity field analysis, the disturbance of the backflow from R2 would affect the airflow field behind the building and then affect the pollutant distribution in R3. When an accidental release happened, if the occupants detected the smell of the hazardous gas and took a typical emergency measure to close the window, the estimated building-averaged $K_{c}$ is smaller than 1/200 of the results obtained under VP1, which is about 0.008. This estimation is based on the infiltration model provided by ASHRAE [45], and a typical residential building has an infiltration rate which is roughly less than 1/200 of the ventilation rate in the presented case scenario. Thus, this extremely low concentration level can be considered as insignificant under closed-window condition.

### 4.4. Impact of Outdoor Source Location on Concentration Field

**Figure 10:**
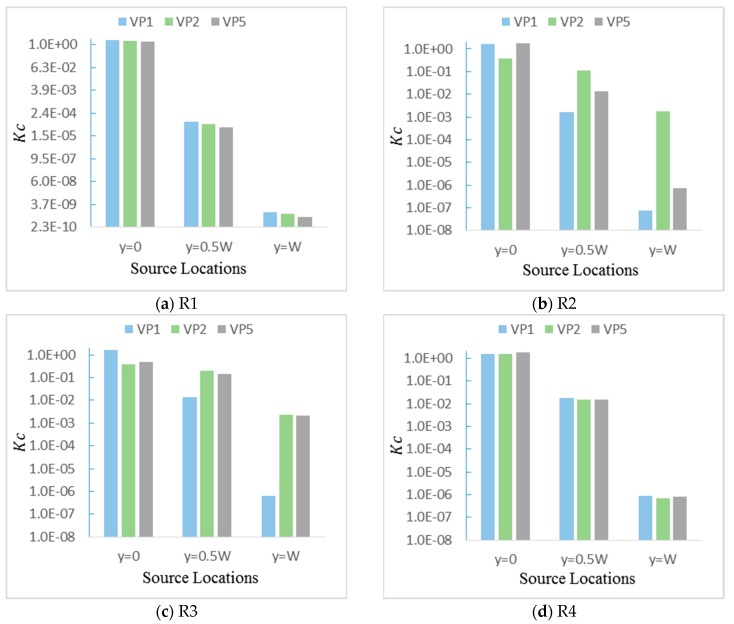
Nondimensional concentration ($K_{c}$) at the z = 1.2 m cross-section in each room for different source locations (y = 0 W, 0.5 W, 1 W). (**a**) R1; (**b**) R2; (**c**) R3; (**d**) R4.

When the pollutant source is located at y = 0 W, the characteristics of the pollutant field in each room under different ventilation paths were analyzed in detail in Section 4.3. Here, the distribution characteristics of the indoor pollutant concentration when the pollutant source is leaked at y = 0.5 W and y=1 W are discussed. It should be noticed that the concentration field may be different when the pollutant source is leaked at y = −0.5 W and y = −1.0 W because the multi-room building is set not to be completely symmetric. As displayed in Figure 10, the indoor concentration value is strongly affected by the source location. It can be obviously seen that the pollutant concentration in the interior space of the building decreases significantly with the increase of the lateral distance from the source point to the building because the approaching wind accelerates the downwind diffusion of the contaminants and inhibits the lateral diffusion of the contaminants when the source location does not directly face the building inlet.

Moreover, the pollutant concentration in the same room can be varied under different natural ventilation paths regardless of the outdoor source location. For R1 and R4, as shown in Figure 10a,d, the differences of the indoor pollutant concentration under the three presented natural ventilation paths are fairly small, despite the location of the pollutant source, because the room in the windward side is more easily affected by the approaching wind, and the relatively large ventilation rate leads to similar indoor pollutant concentrations. While in Figure 10b,c, the differences are nonnegligible in R2 and R3 under the three natural ventilation paths. When the pollutant is leaked at y = 0 W, the pollutant concentrations entering R2 and R3 are the lowest under VP2. However, the pollutant concentrations entering R2 and R3 under VP2 are higher than those under VP1 and VP5 when the pollutant is leaked at y = 0.5 W and 1 W.

### 4.5. Impact of the Source Strength on Human Death Probability

The concentration field could be further examined from a practical point of view using the dose-response model, with respect to the assessment of exposure risk. This study is carried out under the condition that the release velocity of the pollutant source does not affect the airflow field. In accordance with a real situation, when the maximum leakage diameter in the prototype is 1 m and the corresponding exposure time is 30 min, the maximum volumetric gas flowrate of the pollution source is 0.44 m^3^/s, as calculated from Equation (6). The value of the volumetric gas flowrate of more than 0.44 m^3^/s is beyond the scope of this paper. The threshold concentrations corresponding to 1%, 50% and 99% mortality rates are 267 460 and 794 ppm, respectively, as calculated from Equations (11)–(13), respectively. Based on these threshold concentrations and the simulated nondimensional concentrations, the corresponding source strengths that can lead to different mortality rates can be calculated with Equation (10). The details of the relationship between the mortality rates and the corresponding source strengths under five ventilation paths are presented in Table 2, Table 3 and Table 4.

As shown in Table 2 and Table 3, it can be seen that the rooms with the highest and lowest concentrations under different ventilation paths are quite different when H_2_S is accidentally leaked in front of the windward side of the building (y = 0 W). Consequently, the corresponding source strengths calculated based on the highest or lowest concentration can be quite different. Based on different mortality rates, the corresponding source strengths under different ventilation paths are presented. For example, assuming the mortality rate is equal to 1%, the related minimum value of the source strength appears in R2 under VP3 and is 7.31 × 10^−3^ m^3^/s, which is calculated for the highest concentration room. When the volume rate of the source is lower than this value, the mortality rate in the entire indoor environment is lower than 1%. The related maximum value of the source strength appears in R2 under VP2 and is 3.63 × 10^−^^2^ m^3^/s, which is calculated for the lowest concentration room. When the volume rate of the source is higher than this value, the mortality rate in the entire indoor environment is higher than 1%. These differences can reach approximately 4 times and cannot be overlooked. Similar trends can be observed with 50% and 99% mortality rates. Under the same ventilation path, when the corresponding source strength is changed to be 2 times larger, the related mortality rate can be increased from 1% to 99%. In Table 2, it can also be seen that the biggest differences between the two ventilation paths is only 16.7% under the same mortality rate, which appears on R4 under VP2 and on R2 under VP3. In Table 3, the biggest differences between the two ventilation paths can reach 3.3 times under the same mortality rate, which appears on R4 under VP1 and on R2 under VP2.

As displayed in Table 2 and Table 4, the influence of pollutant leakage on the side (y = 0.5 W) of the multiroom building is far less than pollutant leakage in the center (y = 0 W). Under VP2, the differences of the corresponding source strength between leakage from the side and from the center are as high as 7.2 times when the mortality rate in the room is approximately the same. Under VP5, this difference can even reach approximately 11 times. Moreover, R3 gradually becomes the most dangerous room because it is on the leeward side and is relatively closer to the pollutant source. From the perspective of actual exposure assessment, this section quantifies the relationship between the source strength and the mortality rate under different ventilation paths and source locations, which provides an effective method for risk analysis and control after a chemical leakage accident. 

## 5. Conclusions

Reasonable natural ventilation strategies have the advantages of saving energy and ensuring indoor air quality. This paper presents CFD simulations of wind-induced natural ventilation in a single-story multiroom building. For gas-particle flows, the particle inertia is presented by a dimensionless number, Stokes number, and this important dimensionless parameter in particle dynamics determines whether or not they are traveling with the surrounding gas. When the airflow velocities are generally low and the contaminant particles are with small diameters, the Stokes Number for the contaminant particles flow is far less than unity, and the particles will act like gas tracers [46]. Hence, the results shown in Section 4.3 and Section 4.4 will also be helpful in understanding small particle transmission under the presented scenario. The influences of the ventilation path, wall porosity and source location on the indoor air quality are systematically investigated by a validated numerical method. By comparing the velocity and concentration fields inside the building, the present studies have led to the following conclusions:

(1) Two commonly used wall porosities (5% and 10%) were considered in this study, and the effect is not significant under the presented two wall porosities. The effect of wall porosity under a wider range may not be overlooked, which deserve further investigations. The room under the cross-ventilation condition has a much larger $Q^{*}$ value than that of under the single-sided ventilation condition, while the room located on the windward side also has a larger $Q^{*}$ value than that on the leeward side room, regardless of the ventilation path.
(2) The indoor velocity and concentration fields are obviously different under the five natural ventilation paths. In the view of velocity field, VP2 corresponds to the worst ventilation path. However, VP2 corresponds to the best ventilation path in the view of concentration field. Under VP2, the pollutant concentration in the windward room is approximately 4 times that in the leeward room. The single-outlet ventilation path will affect the airflow distribution in the wake area of the building and then the concentration distribution in R3. The room-averaged concentration in R3 leads to the following ranking from the lowest to the highest: under VP2 (taken as the reference value), under VP5 (27.0% higher) and under VP4 (45.9% higher).
(3) The pollutant concentration in the building decreases significantly with the increase of the lateral distance from the source point to the building. The value of the pollutant concentration under VP2 is the lowest when the pollutant is leaked at y = 0 W. However, the pollutant concentrations entering R2 and R3 under VP2 are higher than those under VP1 and VP5 when the pollutant is leaked at y = 0.5 W and 1 W.
(4) To further assess the potential exposure risk to the indoor personnel caused by the leakage of H_2_S, the dose-response model is used to quantify the impact of the source strength on the injury of indoor personnel. Under the same ventilation path, when the source strength is changed to be two times larger, the related mortality rate increases from 1% to 99%. The corresponding source strength is changed by approximately four times when both the highest concentration room and all the rooms reach the same mortality rate.

## Figures and Tables

**Table 1:** Case setup.

Case	Source Location	Ventilation Path	Wall Porosity(%)
1	y = 0 W	VP1	10
2		VP2	10
3		VP3	10
4		VP4	10
5		VP5	10
6		VP1	5
7		VP2	5
8		VP3	5
9		VP4	5
10		VP5	5
11	y = 0.5 W	VP1	10
12		VP2	10
13		VP5	10
14	y = 1 W	VP1	10
15		VP2	10
16		VP5	10

**Table 2:** The corresponding source strengths calculated by the highest concentration room when the pollutant source is leaked at y = 0 W.

Ventilation Path	Room	Concentration (ppm)	Kc	Corresponding Source Strength That Can Lead to Different Mortality Rates (m^3^/s)		
				1%	50%	99%
VP1	R1	7.72 × 10^1^	1.71 × 10^0^	7.79 × 10^−3^	1.34 × 10^−2^	2.32 × 10^−2^
VP2	R4	7.05 × 10^1^	1.56 × 10^0^	8.53 × 10^−3^	1.47 × 10^−2^	2.54 × 10^−2^
VP3	R2	8.21 × 10^1^	1.82 × 10^0^	7.31 × 10^−3^	1.26 × 10^−2^	2.18 × 10^−2^
VP4	R1	7.69 × 10^1^	1.70 × 10^0^	7.81 × 10^−3^	1.35 × 10^−2^	2.32 × 10^−2^
VP5	R2	8.04 × 10^1^	1.78 × 10^0^	7.47 × 10^−3^	1.29 × 10^−2^	2.22 × 10^−2^

**Table 3:** The corresponding source strength calculated by the lowest concentration room when the pollutant source is leaked at y = 0 W.

Ventilation Path	Room	Concentration (ppm)	Kc	Corresponding Source Strength That Can Lead to Different Mortality Rates (m^3^/s)		
				1%	50%	99%
VP1	R4	7.11 × 10^1^	1.57 × 10^0^	8.45 × 10^−3^	1.46 × 10^−2^	2.51 × 10^−2^
VP2	R2	1.66 × 10^1^	3.66 × 10^−1^	3.63 × 10^−2^	6.25 × 10^−2^	1.08 × 10^−1^
VP3	R1	6.06 × 10^1^	1.34 × 10^0^	9.92 × 10^−3^	1.71 × 10^−2^	2.95 × 10^−2^
VP4	R3	2.44 × 10^1^	5.41 × 10^−1^	2.46 × 10^−2^	4.23 × 10^−2^	7.31 × 10^−2^
VP5	R3	2.13 × 10^1^	4.71 × 10^−1^	2.82 × 10^−2^	4.86 × 10^−2^	8.39 × 10^−2^

**Table 4:** The corresponding source strength calculated by the highest concentration room when the pollutant source is leaked at y = 0.5 W.

Ventilation Path	Room	Concentration (ppm)	Kc	Corresponding Source Strength That Can Lead to Different Mortality Rates (m^3^/s)		
				1%	50%	99%
VP2	R3	9.33 × 10^0^	2.06 × 10^−1^	6.44 × 10^−2^	1.11 × 10^−1^	1.91 × 10^−1^
VP5	R3	6.66 × 10^0^	1.47 × 10^−1^	9.02 × 10^−2^	1.55 × 10^−1^	2.68 × 10^−1^
